# Notes on the Method of Preserving Bodies for Dissection at Medical Department, University of Texas, Galveston

**Published:** 1892-06

**Authors:** William Keiller

**Affiliations:** M. D., F. R. C. S. Ed., Professor of Anatomy, Galveston. (Lately Lecturer on Anatomy School of Medicine, Edinburgh.)


DANIEL'S

### Texas Medical Journal.

A Representative Organ of the Medical Profession, and an Exponent of Rational Medicine; devoted to the Organization, Advancement and Elevation of the Profession in Texas.

PUBLISHED MONTHLY.— SUBSCRIPTION $2.00 A YEAR.

Vol. VII. AUSTIN, JUNE, 1892. No. 12.

### Original Articles.

CONTRIBUTED EXCLUSIVELY TO THIS JOURNAL.

*The Articles in this Department are accepted and published with the understading that we are not responsible for, nor do we indorse the views and opinions of the writers by so doing.*

For Daniel's Texas Medical Journal.

### NOTE ON THE METHOD OF PRESERVING BODIES FOR DISSECTING PURPOSES IN USE AT THE MEDICAL DEPARTMENT OF THE UNIVERSITY OF TEXAS.

BY WILLIAM KEILLER, F. R. C. S. ED.,

Professor of Anatomy, Galveston. (Lately Lecturer on Anatomy School of Medicine, Edinburgh.)

[Read before the Galveston Medical Club, April 11, 1892.]

WHEN I came to occupy the chair of Anatomy in Galveston I was told that I would have difficulty in keeping bodies for dissecting; and in the preface to a recent American work on anatomy I read the following: The student's "distaste for the actual labour" of dissecting, "besides being exacting, is associated with much that is revolting and even hazardous to health," a sentence which seems to indicate a state of matters in the average American dissecting room by no means creditable to this age of antiseptics. I have heard also that a supposed difficulty in preserving bodies in this warm Southern climate has been urged as a reason why Texans should choose a school in the colder North as their alma mater. For these reasons, it appears to me, that some account of my methods of preserving bodies may be interesting.

I may say that I have had no difficulty in keeping dissecting room subjects throughout the entire session; that I have specimens now that were dissected in the beginning of the year, and that the same specimens will be preserved for purposes of revision by the students that join us next session; that students are allowed to dissect as leisurely as they may desire, and can take time to examine every structure with the utmost care, spending three months at a single part if necessary; and that my dissecting-room has no odor whatever. I firmly believe that a surgeon might, without the slightest risk, go from dissecting one of my subjects straight to a laparotomy with no more than the usual precautions.

While lecturing on anatomy at Edinburgh, I had to be in a position to leave the dissecting room for a confinement at any moment. To do this is contrary to professional opinion; but I resolved to keep my subjects and my rooms in such a manner that I should involve my patients in no risk, and though I had frequently to leave the dissecting room for a forceps case and once for a case where intra-uterine manipulation was necessary, I never had an untoward result.

My plan there was to inject the body as soon as I received it with four ounces of corrosive sublimate dissolved in 180 ounces methylleted spirits of wine, the injection being done by fluid pressure from a height of four feet through the common femoral artery. The result was that in less than half an hour all the tissues were fixed and all the body turned white by the mercurial salt. It was then only necessary to paint the body over with glycerine and carbolic acid (1 to 10) and wrap it in a sheet dipped in 1 to 500 perchloride of mercury solution in water and then in oil cloth and the body would keep indefinitely with very little attention.

Here, I have found the following method answer best, and though I have only had a winter's experience in Galveston, I would have no hesitation in undertaking to keep the dissecting room sweet, even in the moist heat of our sub-tropical summer:

As soon as possible after death, expose the right common carotid artery and right internal jugular vein by a skin incision along the anterior border of the sternomastoid, extending upwards for two inches from the sternum, and another across the muscle from the lower end of the first incision; reflect this angular flap with platysma, etc., and cut through the lower end of the sternomastoid, reflecting it upwards sufficiently to expose the sheath of the great vessels.

Open the internal jugular vein and pass a straight glass tube of one-fourth inch bore (or larger) into the right auricle of the heart; tie in the tube and suck all the blood out of the heart by attaching the tube to an aspirator bottle exhausted by the usual aspirator syringe (such as Potain's). A very good aspirator in hard rubber is sold by the Egyptian Chemical Company, St. Louis, Mo. In doing so, elevate, in order, the arms, head, legs, and trunk as much as possible above the level of the heart. Now remove the aspirator bottle and connect the tube from the auricle with an empty vessel by a piece of rubber tubing. Insert a glass T tube into the carotid artery. This can be done by an incision ¾ inch long if the T tube have each cross limbs as short as possible, viz: ½ inch with a constriction for tying in ¼ inch from the end. Tie each end of the cross limbs into the vessel, thus one end will carry the injected fluid towards the head, the other towards the heart. Inject the arteries of the body with the following preservative:

| | |
|---|---|
| Perchloride of Mercury | ½ lbs. |
| Chloride of Zinc | 3 lbs. |
| Strong Hydrochloric Acid | 2 oz. |
| Water | 4 gallons. |

Four gallons of the fluid is to be prepared and placed in a stoneware jar provided with a glass or wooden stop-cock; the jar being a foot or more above the body, 10 feet of rubber tubing is to be attached to the stop-cock, the fluid turned on, and when all air is driven out of the tube it is to be connected to the T tube. The jar is now to be run up to the roof by a rope and pulley arrangement, and the continuous pressure thus obtained causes the preservative to thoroughly permeate the vessels. Soon, almost pure blood, and then blood more and more mixed with preservative will flow from the internal jugular vein, and this is to be allowed to run till fairly clear. Elevate again in turn the legs, arms, head, and trunk, holding them up so long as by so doing you increase the proportion of blood in the fluid pouring from the jugular vein. When this fluid runs fairly clear clamp the rubber tube so as to confine the rest of the injection in the veins and thus produce more pressure. If the injection be successful every particle of skin on the body should be turned white. The feet are most difficult to inject; but if they do not turn white readily raise the trunk to a sitting posture; the body is most conveniently handled if lying on the floor of the preparing room. Now, in temperate climates it will be sufficient to rub the body over with glycerine or carbolic acid (1 in 15), especially between the toes and similar crevices to prevent fungi growing on the skin, and wrap it in a sheet steeped in methyllated spirits of wine (wood alcohol) or 1 to 500 watery solution of corrosive sublimate, or 1 20 arseniate of soda solution (the last perhaps is the best) and keep it in a fairly tightly covered tank; but in this climate I find it advisable to keep the body immersed in some antiseptic. At present I am using a saturated solution of sodium chloride with 1 to 500 arsenide of soda, which gives satisfactory results; but sodium chloride and chloride of zinc, or sodium chloride and perchloride of mercury might be better. The mercury has a tendency to blacken the tissues, I suppose by precipitating slowly black oxide or metallic mercury. I shall try the chlorides of sodium and zinc at an early date. The necessity is a cheap antiseptic which will not macerate and separate the stratum corneum, will thoroughly protect against fungi, and yet leave the skin quite pliable. The longer the body lies in the fluid the better will it keep when brought to the dissecting table.

The tubes should be corked and left in the vessels till the red injection is thrown into the arteries. This should not be done for at least some days after the preservative fluid has been used.

I regret that I am not in a position to speak definitely about a colored injection mass. Plaster of paris, one of the oldest, is clean, and easily used after a very little practice, but it forms a hard, rigid, brittle mass in the larger vessels, which prevents them bending, and renders them apt to tear; objections more appreciated by the careful dissector than they appear valid on paper.

A mass formed of red and white lead, boiled linseed oil, turpentine and gold size, etc., is excellent in many ways, and I shall try it next year. My past experience of it was in bodies injected with spirit or a preservabric rich in glycerine, and it frequently did not solidify thoroughly in the larger vessels, due, I think, to the glycerine or spirit. Otherwise, it was very satisfactory, and I think it will give good results with bodies injected with aqueous solutions only.

In colder climates, fine glue or gelatine masses give excellent results; but so far, I am not quite pleased with them here, though the little inconveniences I am alluding to create very little discomfort. We have got on very well with gelatine masses this winter, still I am not quite satisfied. Next year I hope to report almost perfect results, but if any of my readers wish a mass which will give fair satisfaction, the following may be used. In all colored injections be careful not to use aniline colors in any form, as they stain the tissues. Steep fine glue (broken small) for one day, in as much water as covers it, heat next day with a little water in a vessel set in boiling water, taking care not to boil the glue. Allow it to cool so as to see how it will set, and the following day add water or glue, as may be necessary to render it fairly firm. The proportion will differ according to the season. A little carbolic acid added will keep the jelly till required. Melt this, add red lead enough to color it sufficiently, run it through a fine wire strainer, and with a syringe throw it into the arteries as hot as the hand can bear. If this be done quickly, it will flow freely into all the fine vessels. While injecting the red fluid the vein should be unclamped, but reclamped should any red solution reach the veins. The glue is clean to work with should any vessel leak, and gives sufficient prominence to the arteries. About forty ounces will be required for a body.

When the subject is put on the table, it should be wrapped in a sheet wrung out of arsenate of soda (1 in 20) solution, and then in oilcloth. This is carefully replaced whenever the students finish dissecting. The various parts, when removed from the body, are handed over to the care of the dissector, who is responsible for their keeping. He must wrap the parts in carbolic acid and glycerine (1 to 15), rubbing the glycerine thoroughly over the parts exposed in each day's dissection, and keeping his dissection wrapped in oilcloth, with his card tied to it.

Should fungi develop in any part, it must be immersed in the bath.

The only objection to the fluid I at present use as a preservative, is that it precipitates albumen, and turns the tissues white; but this appears to me of very minor importance, compared with its efficiency as a preservative, and especially in view of the following result, whicb I consider one of great value, and which cannot be attained without altering the color of the tissues; I mean the hardening effect on the muscles, but especially on the heart, liver, spleen, kidneys and adrenal bodies. In my subjects the muscles retain their form without being so hardened as to impede dissection, and when one opens the cavities, the result is perfect. The liver stands out with all its five surfaces, its depressions and protuberances fixed in their natural coutour instead of spreading flat out like a dead jelly-fish, and the spleen keeps its borders, impressions and surfaces perfect, instead of present- a mere pulpy mass for the student's edification; the kidneys, adrenals and pancreas retain all their characteristics, and the heart presents a base, apex, and three surfaces, instead of flapping out on the table the shapeless organ that has given rise to its description as possessing two surfaces and two borders.

I shall have something to say in a future paper on the description of these organs as we find them when hardened in situ; I will only say in the meantime that my results are confirmatory of those obtained by Prof. His, and illustrated by his excellent models.

I would say in closing, that the post mortem room may be revolting and injurious to health; a properly kept dissecting room should be neither.

